# Crystal structure of the first eukaryotic bilin reductase *Gt*PEBB reveals a flipped binding mode of dihydrobiliverdin

**DOI:** 10.1074/jbc.RA119.009306

**Published:** 2019-07-31

**Authors:** Johannes A. Sommerkamp, Nicole Frankenberg-Dinkel, Eckhard Hofmann

**Affiliations:** ‡ Protein Crystallography, Faculty of Biology and Biotechnology, Ruhr University Bochum, 44801 Bochum, Germany; § Department of Biology, Microbiology, Technical University Kaiserslautern, 67663 Kaiserslautern, Germany

**Keywords:** algae, biosynthesis, crystal structure, photosynthetic pigment, enzyme mechanism, ferredoxin-dependent bilin reductase, phycoerythrobilin, radical enzyme, regiospecificity, tetrapyrrole

## Abstract

Phycobilins are light-harvesting pigments of cyanobacteria, red algae, and cryptophytes. The biosynthesis of phycoerythrobilin (PEB) is catalyzed by the subsequent action of two ferredoxin-dependent bilin reductases (FDBRs). Although 15,16-dihydrobiliverdin (DHBV):ferredoxin oxidoreductase (PebA) catalyzes the two-electron reduction of biliverdin IXα to 15,16-DHBV, PEB:ferredoxin oxidoreductase (PebB) reduces this intermediate further to PEB. Interestingly, marine viruses encode the FDBR PebS combining both activities within one enzyme. Although PebA and PebS share a canonical fold with similar substrate-binding pockets, the structural determinants for the stereo- and regiospecific modification of their tetrapyrrole substrates are incompletely understood, also because of the lack of a PebB structure. Here, we solved the X-ray crystal structures of both substrate-free and -bound PEBB from the cryptophyte *Guillardia theta* at 1.90 and 1.65 Å, respectively. The structures of PEBB exhibit the typical α/β/α-sandwich fold. Interestingly, the open-chain tetrapyrrole substrate DHBV is bound in an unexpected flipped orientation within the canonical FDBR active site. Biochemical analyses of the WT enzyme and active site variants identified two central aspartate residues Asp-99 and Asp-219 as essential for catalytic activity. In addition, the conserved Arg-215 plays a critical role in substrate specificity, binding orientation, and active site integrity. Because these critical residues are conserved within certain FDBRs displaying A-ring reduction activity, we propose that they present a conserved mechanism for this reaction. The flipped substrate-binding mode indicates that two-electron reducing FDBRs utilize the same primary site within the binding pocket and that substrate orientation is the determinant for A- or D-ring regiospecificity.

## Introduction

**Figure 1.**
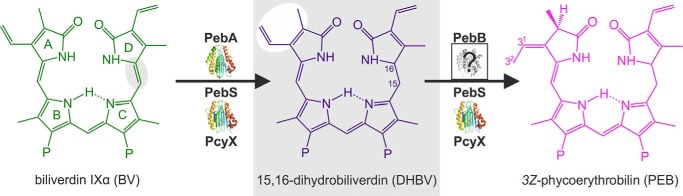
**Different routes of PEB biosynthesis.** Specific FDBRs convert BV via the intermediate DHBV to the pink pigment PEB. PebA converts the substrate BV via a two-electron reduction to the intermediate DHBV, which is subsequently reduced by PebB to the final product PEB. Whereas PebA and PebB work in tandem, PcyX as well as PebS combine this dual enzyme system in a single protein and are capable of catalyzing the whole four-electron reduction of BV via DHBV to PEB.

Phycobilins are open-chain tetrapyrroles involved in light-harvesting and light-sensing of cyanobacteria, cryptophytes, and red algae. Their biosynthesis starts with the cleavage of a heme macrocycle by a heme oxygenase, yielding the first open-chain tetrapyrrole pigment biliverdin (BV)^2^ IXα (1). BV IXα is then the substrate for many members of the ferredoxin-dependent bilin reductases (FDBRs) that catalyze a regio- and stereospecific reduction of the tetrapyrrole pigment. The FDBR family can be divided into members that catalyze a formal two-electron reduction and members catalyzing a four-electron reduction. However, all FDBRs seem to adopt the same overall fold: an α/β/α-sandwich with the active site being on top of the central β-sheet (2–6). Thus far, the most interesting and diverse pathway is the biosynthesis of the pink pigment phycoerythrobilin (PEB; Fig. 1). This phycobilin is mainly used by marine cyanobacteria, red algae, and cryptophytes in their phycobiliprotein light-harvesting structures (7, 8). Although all of these organisms require two FDBRs to convert BV to PEB, marine phages encode enzymes that combine these activities within one enzyme (9, 10). The biosynthesis is initiated through the binding of BV by 15,16-dihydrobiliverdin:ferredoxin oxidoreductase (PebA); once bound, the substrate is protonated by a highly conserved aspartate residue located on the central β-sheet of the enzyme. Following electron transfer from the electron donor ferredoxin, a substrate radical is formed that can be easily detected by EPR spectroscopy (5). Subsequent protonation and proton coupled electron transfer result in the stereospecific reduction of the C15–C16 double bond of BV yielding 15,16-DHBV. This biosynthetic intermediate is then transferred via proximity channeling (11, 12) to PEB:ferredoxin oxidoreductase (PebB). PebB is thus far the only FDBR member that cannot use BV as a substrate. In contrast the semireduced 15,16-DHBV is employed. Upon binding, this substrate is also protonated by the central aspartate. However, during the reaction, a second aspartate residue gets involved and likely serves as a proton donor for the proton coupled electron transfer to the A-ring of the tetrapyrrole molecule. This reaction catalyzed by PebB is a formal reduction of the 2,3,3^1^,3^2^-dien system of the A-ring of 15,16-DHBV. A similar reduction is catalyzed by the plant FDBR phytochromobilin synthase (HY2), which uses BV as a substrate and catalyzes the two-electron reduction to phytochromobilin (13–15). An A-ring reduction is furthermore catalyzed by the four-electron reducing FDBRs, phycocyanobilin:ferredoxin oxidoreductase (PcyA), which catalyzes the conversion of BV to PCB via a bound intermediate 18^1^,18^2^-DHBV, and phycoerythrobilin synthases PebS/PcyX that both catalyze the reduction of BV via 15,16-DHBV to PEB (6, 9, 10, 16, 17).

All four-electron reducing FDBRs have in common that the A-ring reduction is the second reduction step, with each using a specific substrate intermediate. Although many FDBR crystal structures with and without bound substrates have been solved in the last 13 years, one of an FDBR specifically reducing the A-ring was still missing. Here we solved the crystal structure of the PebB homolog PEBB (nuclear encoded proteins in eukaryotes are denoted with capital letters by convention) from the eukaryotic cryptophyte alga *Guillardia theta*. This organism evolved by secondary endosymbiosis and employs soluble phycobiliproteins in the thylakoid lumen of the chloroplast (7, 18). The nucleus encoded FDBRs *Gt*PEBA and *Gt*PEBB were previously identified and initially characterized (19). The new crystal structures of *Gt*PEBB presented herein gave some unexpected insights into the A-ring reduction of phycobilins. They thereby contribute to a better understanding of the underlying catalytic mechanism.

## Results and discussion

### Structure solution of GtPEBB

The X-ray structure of selenomethionine-labeled *Gt*PEBB was determined by single-wavelength anomalous dispersion. We were able to build and refine an initial atomic model for the majority of the protein, but density for the N-terminal extension unique to eukaryotes was absent. Therefore, we designed an expression construct for a truncated version of *Gt*PEBB lacking the first 83 amino acids, which were annotated as chloroplast transit peptide by ChloroP (20). Incidentally, this construct contains the full FDBR domain and reflects the mature *Gt*PEBB (m*Gt*PEBB). Crystals of m*Gt*PEBB diffracted slightly better, showing the same space group (P6_2_) and almost identical unit cell parameters. The structure of m*Gt*PEBB was refined at 1.9 Å resolution to an *R* factor of 21.1% and a free *R* factor of 23.2% (Table 1). The first three residues (two residues of the linker peptide used in the expression construct, one residue of the mature protein) and residues 45–48 and 147–151 have been omitted because of missing or weak density.

**Table 1:** **Data collection and refinement statistics** The data in parentheses represent values for the highest resolution shell. ESRF, European Synchrotron Radiation Facility; SLS, Swiss Light Source.

	*Gt*PEBB	m*Gt*PEBB	m*Gt*PEBB-DHBV
	Anomalous data set (gtpebb-s10)	Native data set (gtpebb70-4-5)	Native data set (gtbdh31)
**Data collection**			
Beamline	ESRF ID30B	SLS X10SA	SLS X10SA
Resolution (Å)	42.88, 2.5 (2.59, 2.5)	42.82, 1.9 (1.968, 1.9)	40.63, 1.65 (1.71, 1.65)
Cell parameters			
*a*, *b*, *c* (Å)	109.54, 109.54, 48.07	109.6, 109.6, 47.98	47.69, 77.69, 80.94
α, β, γ (°)	90, 90, 120	90, 90, 120	90, 91.98, 90
Space group	P 62	P62	P 1 21 1
Wavelength (Å)	0.97625	1.006	0.97898
No. of observations	365,817 (37,827)	490,525 (14,730)	477,875 (44,741)
No. of unique reflections	11,471 (1133)	25,797 (2492)	69,636 (6805)
Completeness (%)	98.95 (98.87)	98.70 (97.04)	98.12 (96.66)
Multiplicity	31.9 (33.4)	19.0 (5.9)	6.9 (6.6)
Average *I*/σ*I*	17.98 (1.99)	15.3 (1.41)	9.71 (1.00)
CC_½_	0.999 (0.66)	0.999 (0.504)	0.998 (0.445)
Wilson B-factor	51.79	34.5	20.89
*R*_merge_		0.1849 (1.455)	
*R*_meas_	0.2264 (2.273)	0.1889 (1.595)	0.1414 (1.941)
**Phasing**			
Selenium sites	10		
Figure of merit	0.265		
**Refinement**			
*R*_work_/*R*_free_ (%)		21.1/23.2	18.22/21.09
Root-mean-square deviation bond length (Å)		0.006	0.003
Root-mean-square deviation angles (°)		0.78	0.66
Ramachandran favored		98.05	98.26
Ramachandran outlier		0	0
Average B-factor (Å^2^)		40.42	28.86
Protein (Å^2^)		39.98	27.12
Ligands (Å^2^)		76.17	39.98
Solvent (Å^2^)		41.91	39.17
**No. of atoms (without riding hydrogens)**		2394	5164
Protein		2206	4427
Water		168	610
Sulfate ion		20	25
Pentaethylenglycol			16
Dihydrobiliverdin			86
PDB code		6QWQ	6QX6

The substrate-free model of m*Gt*PEBB was used to determine the structure of the m*Gt*PEBB–DHBV complex, which crystallized in space group P2_1_ with two molecules in the asymmetric unit. Both chains showed clear density for DHBV already after molecular replacement, even though chain A had overall higher B-values and somewhat lower occupancy of the substrate. Electron density for the vinyl groups defined the orientation of the substrate in both chains.

### Mature mGtPEBB retains full activity

**Figure 2.**
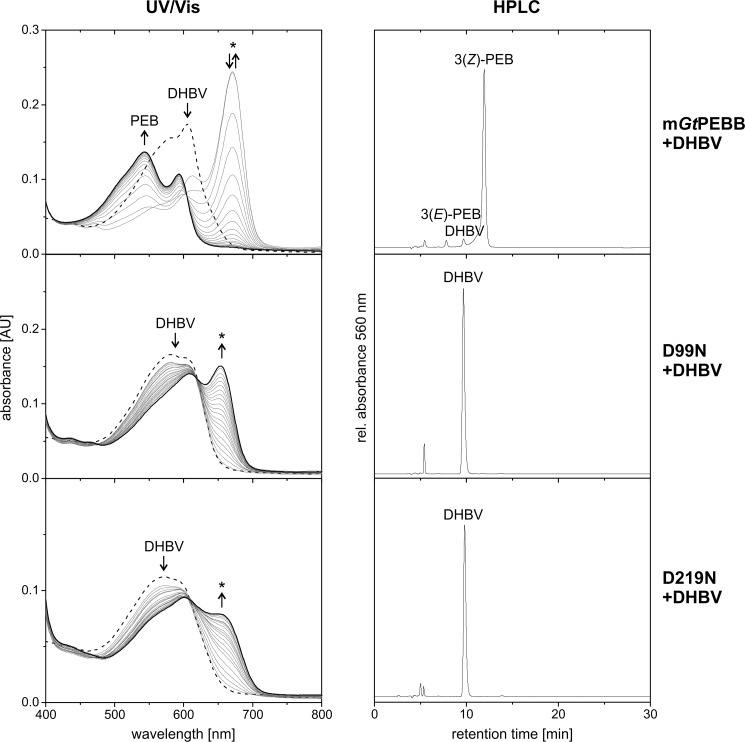
**m*Gt*PEBB converts DHBV to PEB like the full-length protein, and conserved aspartate residues are crucial for the activity.** The reactions of m*Gt*PEBB and variants were monitored by UV-visible (*UV/Vis*) spectroscopy and HPLC analysis of the reaction products. UV-visible spectra were taken every 30 s for 10 min. The first spectrum represents the DHBV–protein complex and is shown as a *dashed line*. The final spectrum of the reaction is shown in *bold. Arrows* indicate the development of the spectra during the reaction, and possible radical intermediates are marked with *asterisks*. The reaction products after 10 min were analyzed with HPLC monitoring the absorbance at 560 nm.

*Gt*PEBB has been found to show *bona fide* 15,16-DHBV:ferredoxin oxidoreductase activity (19). To make sure that mature m*Gt*PEBB lacking the chloroplast transit peptide is equally active, we utilized an anaerobic enzyme assay with analysis of UV/VIS absorption followed by HPLC analysis (21). Upon addition of substrate DHBV, a red shift of the absorption spectrum indicated efficient binding to m*Gt*PEBB (Fig. 2). Immediately after starting the reaction, a decrease of the m*Gt*PEBB–DHBV absorption maximum at 605 nm combined with a strong increase at 670 nm was observed. During the reaction, the absorbance at 670 nm decreased, and product formation was indicated by an increase of the absorbance at 543 nm. The long wavelength absorbance at 670 nm results from the accumulation of substrate radical intermediates and was observed for the full-length protein as well (19). Based on HPLC analysis, the final product is 3*Z*-PEB with only trace amounts of 3*E*-PEB and residual DHBV (Fig. 2). This is in accordance with previous results and the postulate that all FDBRs only produce the 3*Z*-isomer. Presence of small amounts of 3*E*-PEB, the more stable isomer, can be explained by conversion from 3*Z* to 3*E* because of assay work-up conditions (17). This analysis shows that the mature m*Gt*PEBB contains the enzymatically active core of the protein and that the first 83 amino acids of *Gt*PEBB are not essential for the conversion of DHBV to PEB.

### Overall structure of GtPEBB

**Figure 3.**
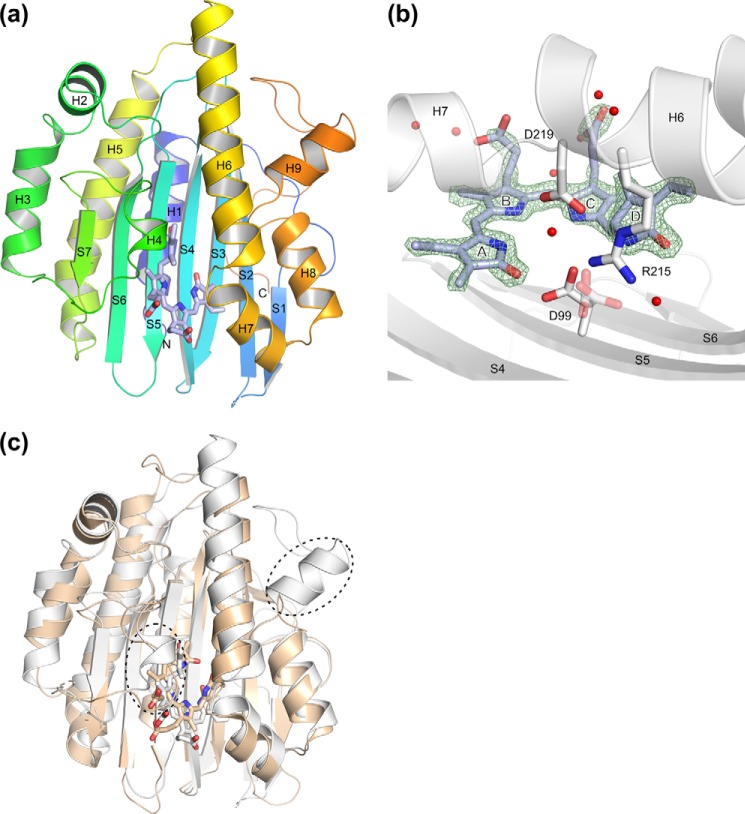
*a*, X-ray structure of m*Gt*PEBB–DHBV (PDB code 6QX6, chain B). Protein is *rainbow-colored* from *blue* (N terminus) to *red* (C terminus), and the substrate DHBV is shown as *purple sticks. b*, active site of m*Gt*PEBB–DHBV. DHBV (*purple*) and residues critical for the PebB reaction are represented as *sticks*. Water molecules in 4 Å distance to DHBV are shown as *red spheres*. A simulated annealing OMIT-map for DHBV is shown as a *green mesh*, contoured at 1.6 σ. *c*, superposition of m*Gt*PEBB–DHBV (PDB code 6QX6, chain B) shown in *light gray* and PebA from *Synechococcus* sp. WH8020 (PDB code 2X9O) colored *beige*. Structural differences discussed in the text are highlighted by *dashed ovals*.

The eukaryotic m*Gt*PEBB is a globular single domain protein that shows the characteristic FDBR α/β/α-sandwich fold with a central antiparallel β-sheet, flanked by α-helices (Fig. 3*a*). DHBV is bound in a niche formed by the central β-sheet on the proximal and by helices H6/H7 on the distal side of the substrate (Fig. 3*b*). When comparing the substrate-free and the substrate-bound *Gt*PEBB structures, only minor conformational changes are observed upon DHBV binding. Both models superimpose nearly perfectly, showing a root-mean-square deviation of 0.31 Å (PyMOL super, 217 Cα atoms). Slight side-chain movements of several residues lining the substrate-binding pocket are observed (Fig. S1). Substrate binding mostly leads to a stronger coordination of the mobile residues between helix H3 and β-strand S7, forming the “lid” of the active site. These residues could not be modeled reliably in the substrate-free structure but showed defined density in the substrate-bound structure.

### Coordination of substrate DHBV

A prominent feature of the active site is the Asp-99/Asp-219 pair conserved in most FDBRs (Fig. 3*b*). Although Asp-219 coordinates a central pyrrole water molecule, Asp-99 is in direct contact with the substrate and coordinates the polar pyrrole nitrogen of the D-ring and the carbonyl oxygen of the A-ring. In our crystal structure, Asp-99 had to be modeled in alternate conformations. Interestingly, computational calculations with a modified PROPKA algorithm showed a shift for the p*K_a_* from below 7 for the two conformations rotated away from the A-ring to above 8 for the conformation in coordinating position toward the carbonyl oxygen (Conformer B, highlighted in Fig. 3*b*). At neutral pH, the latter conformation therefore would have a higher probability to be protonated. This is consistent with the red shift of the absorbance maximum during DHBV binding at neutral pH. In other FDBRs, such a bathochromic shift is attributed to a protonated, positively charged substrate molecule, requiring an adjacent proton donor (16, 22). Titrating the m*Gt*PEBB–DHBV complex from pH 7.5 to pH 8.5 resulted in a reversible hypsochromic shift consistent with a deprotonation of Asp-99 (data not shown). Because the m*Gt*PEBB–DHBV crystals were grown at pH 8.5 and appeared in similar purple color, the observed alternate conformations most likely reflect this deprotonation of Asp-99. Therefore, we propose the highlighted conformer of Asp-99 as the catalytically active ground state, most likely in a protonated form in the productive enzyme–substrate complex. At the same time, the flexibility of Asp-99 could also be a mechanistic requirement for catalysis as described for PcyA, even though both enzymes differ in their respective catalytic mechanism (22). Notably, a neutron scattering study of PcyA showed protonation of the corresponding conformer in close contact to the D-ring carbonyl in the substrate-bound ground state of the enzyme. A second conformer more directed at the pyrrole nitrogens (and an axial water molecule) was found in deprotonated state (23).

### Structural relation to other FDBR

When comparing m*Gt*PEBB–DHBV to the other structurally known FDBR, it superimposes best to PebA from *Synechococcus* sp. WH8020 (PDB code 2X9O) with a low root-mean-square deviation of 1.54 Å (PyMOL super, 186 Cα atoms) (Fig. 3*c* and Table S1). This agrees with the placement of PebBs in a phylogenetic tree close to PebAs (6).

*Gt*PEBB possesses a C-terminal extension (Fig. S2), which forms helix 9 and a long loop (23 amino acids) not present in most other FDBR structures (except HY2 from *A. thaliana*). The largest structural differences to PebA are observed for residues 149–151 (*Gt*PEBB numbering), which are part of the lid of the active site and form a short helix in *Gt*PEBB (Fig. 3*c*). In contrast, no secondary structure elements can be observed in the corresponding stretch of PebA or in PebS (4), whereas in PcyA an even longer helix is found (2). Currently, no mechanistic rationales for this diversification of different FDBRs are apparent. Nevertheless, the more unstructured lid regions are expected to become more readily disengaged, thereby allowing easier access to the active site.

### Aspartate pair is critical for GtPEBB activity

Essential for the conversion of DHBV to PEB by PebB from *Synechococcus* sp. WH8020 is the aspartate pair Asp-107/Asp-231 (5). As discussed above, in m*Gt*PEBB the corresponding residues Asp-99/Asp-219 are indeed integral in substrate binding. We therefore investigated the enzymatic activity of the variants D99N and D219N in m*Gt*PEBB (Fig. 2). Loss of the carboxyl function at these positions caused a blue shift of the maximum of the DHBV-binding spectrum. Over time the absorbance at 655 nm increased slowly, which is most likely due to the accumulation of a substrate radical intermediate as observed for the corresponding variants of the cyanobacterial PebB (5). Accordingly only the substrate DHBV was detected by HPLC. Therefore, both Asp-99 and Asp-219 are essential for catalytic activity of eukaryotic *Gt*PEBB and reduction of DHBV to PEB.

### A flipped binding mode of DHBV

**Figure 4.**
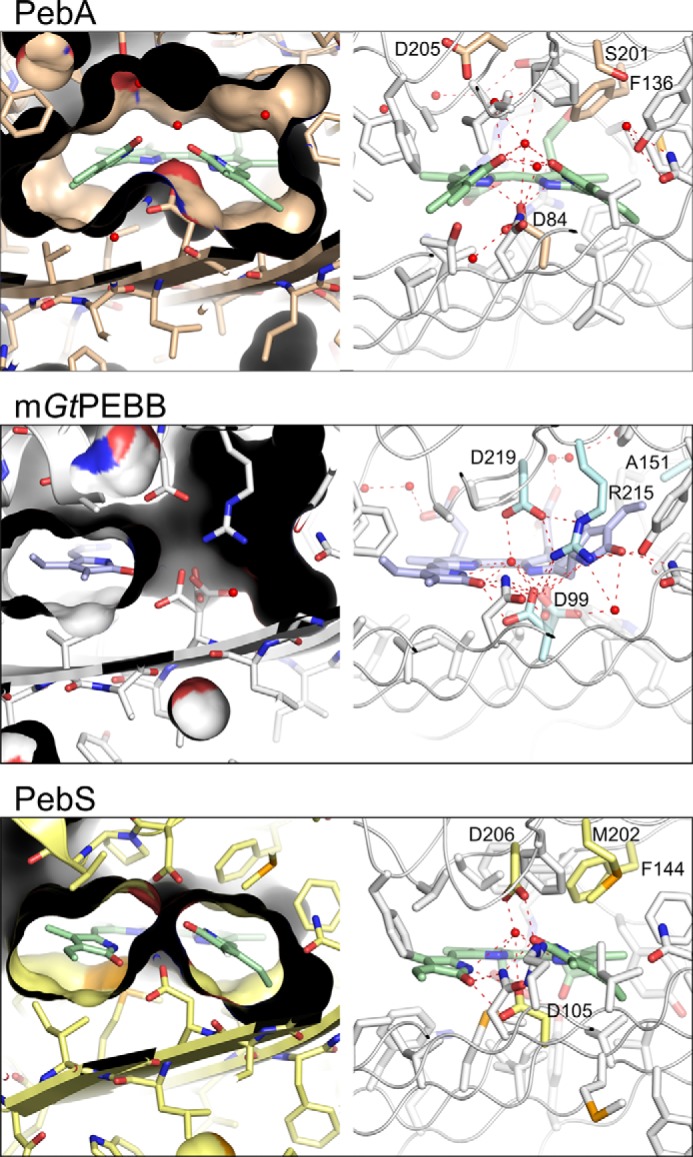
**Active sites of PebA, PebB, and PebS enforce different substrate conformations.** Active sites of PebA (PDB code 2X90), m*Gt*PEBB (PDB code 6QX6, chain B), and PebS (PDB code 2VCK, chain C) with their specific substrate BV (*green*) and DHBV (*purple*) are shown in *stick* representation. BV of PebA and PebS is oriented with the D-ring on the *left* and the A-ring on the *right*, whereas DHBV bound to PebB in a flipped mode is oriented with the D-ring on the *right* and the A-ring on the *left*. Water molecules are shown as *red spheres. Left panels*, the molecular surfaces of the proteins demonstrate the different shapes of the binding niches. *Right panels*, detailed view of the binding niche. Residues in 4.5 Å distance around the substrate are represented as *sticks*; additionally residues discussed in the text are highlighted in *color* and labeled accordingly. *Red dotted lines* represent polar contacts. For stereo views of the binding pockets see Fig. S3.

The basic geometry of the active site of *Gt*PEBB is similar to PebA and all other structurally solved FDBRs (Fig. 4 and Fig. S3). Mostly hydrophobic residues of the central seven-stranded β-sheet build the proximal basis for the binding niche. Asp-99, which is a critical residue in all FDBRs, is located on strand S4. The distal side of the substrate is covered by the two helices H6 and H7 (PebA: H5/H6, PebS: H3/H4, PcyA:H7/H8), which are connected by a short loop containing the critical Asp-219, relevant for those FDBRs catalyzing the reduction of DHBV to PEB.

The Asp-99/Asp-219 pair is structurally conserved in most FDBRs. The corresponding residues in PebB from *Synechococcus* sp. WH8020 and PebS from the cyanophage PSSM2 were identified as catalytically relevant and show the same orientation toward the substrate center (5, 17). For PebA only the homolog of Asp-99 (Asp-84) was found to be essential for catalytic activity (5). The homolog of Asp-219 (Asp-205) is not essential and is rotated out of the active site.

The binding niches of PebA, *Gt*PEBB, and PebS impose spacial restraints on the substrate molecules. For PebA and PebS (both with bound BV), these result in different substrate conformations and relative positions to the catalytic aspartates. One has to note that especially in the case of PebS, variations of these binding conformations have been observed even within one crystal structure, therefore highlighting an increased flexibility needed for the four electron reduction (4).

The most surprising result of the structural analysis of the binding pocket of *Gt*PEBB is the orientation of the bound substrate DHBV. Based on previous studies and homology models of PebB, it was postulated that PebB binds DHBV analogous to the binding of BV in PebA/PebS. Therefore, the β-sheet aspartate (Asp-107, PebB *Synechococcus* sp. WH8020 numbering) was proposed to ensure the stereospecific protonation of C2 at the A-ring, resulting in the *R* configuration. Asp-231 was believed to be involved in direct coordination of the substrate DHBV to support the protonation by Asp-107 (5).

Contrary to this hypothesis, we identified a flipped binding orientation of DHBV in *Gt*PEBB based on strong density for the vinyl groups (Figs. 3*b* and 4 and Fig. S4). This results in an inverted positioning of the A- and D-rings. In addition, although the ligand adopts a planar conformation with three of its pyrrole rings, one pyrrole ring is tilted out of the plane. Only the methine bridge with the C15–C16 single bond between rings C and D (specific for DHBV) supports such a rotation, thereby further corroborating our assignment. This orientation is not compatible with the proposed reaction mechanism outlined above, because the central aspartate now is positioned on the wrong side of the A-ring.

We therefore analyzed the binding pocket to identify structural factors governing this new substrate-binding mode and giving insights into the mechanism of A-ring reduction within PebB. Specific to PebB is the strictly conserved Arg-215, which strongly modifies the shape of the binding pocket with its long positive charged side chain when compared with PebA and PebS (Fig. 4 and Fig. S3). This residue is integrated into a hydrogen-bonding network to several surrounding residues conserved among the members of the PebB family. Compensating the narrowing of the niche, an aromatic residue present in most FDBRs (Phe-136 in PebA) is replaced by a small residue in PebBs (Ala-151 in *Gt*PEBB; Fig. S2). This exchange provides enough space to fit in the tilted D-ring of DHBV.

### Arg-215 is essential for catalytic activity

**Figure 5.**
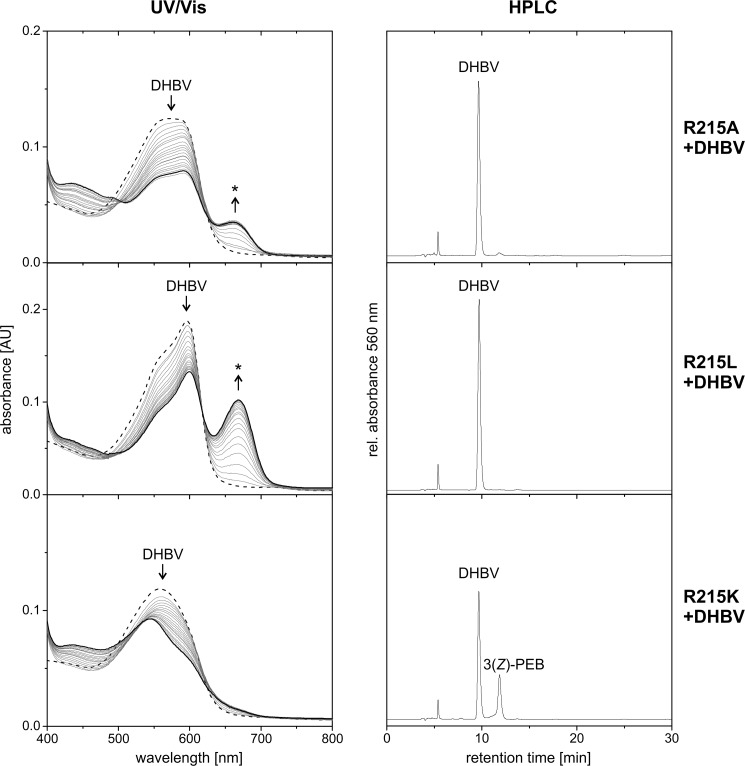
**Arginine 215 is a critical residue for the *Gt*PEBB reaction.** The reactions of m*Gt*PEBB variants were monitored by UV-visible (*UV/Vis*) spectroscopy and HPLC analysis of the reaction products. UV-visible spectra were taken every 30 s for 10 min. The first spectrum represents the DHBV–protein complex and is shown as a *dashed line*. The final spectrum of the reaction is shown in *bold. Arrows* indicate the development of the spectra during the reaction, and possible radical intermediates are marked with *asterisks*. The reaction products after 10 min were analyzed with HPLC monitoring the absorbance at 560 nm.

To further corroborate the role of Arg-215 for the function of PebB, we created several variants at this position. Changing the positively charged side chain to one with hydrophobic character (R215A, R215L) led to a complete loss of activity (Fig. 5). Prominent peaks of DHBV were detected by HPLC for both variants, and only trace amounts of PEB were detected for R215A. The starting spectrum after incubation of R215A with DHBV was drastically different from the WT spectrum and more similar to DHBV in solution. In contrast, the binding spectrum of R215L was very similar to the WT spectrum with a slight shift of the maximum to shorter wavelength. The length of the side chain at this position and the resulting shape of the binding pocket probably are important factors for correct binding of DHBV. Both R215A and R215L most likely stabilize a radical intermediate, as indicated by the prominent absorbance peak ∼670 nm (Fig. 5, marked by an *asterisk*). Replacing Arg-215 with lysine maintained side chain charge and size to some extent but resulted in reduced catalytic activity. Based on the HPLC elution profiles, the amount of produced PEB was only approximately one-quarter of the WT. Interestingly, similar to R215A, this variant showed very ineffective binding as judged by the spectral shift of DHBV absorption. No absorption in the region from 670 to 680 nm was detected for R215K. Because we have to assume that the variant still acts via a radical intermediate, its formation has become rate-limiting. In contrast, in the WT reaction, the rate-limiting step is the conversion of the radical intermediate to the final product PEB, therefore explaining the transient radical accumulation.

It is somewhat puzzling that different variants do not show the classical shift in absorption of the substrate upon incubation yet retain significant catalytic activity. Because this spectral shift can be reverted by a pH shift in the WT enzyme (as discussed above), it is not a precise indicator of binding itself but also requires the correct protonation state of the binding niche.

One major function of Arg-215 is the variation of the shape of the active site by its long side chain as compared with PebA, enforcing the flipped binding mode of DBHV. A second function is the strong coordination of the catalytically important residue Asp-219 by a salt bridge, present in both the substrate-free and DHBV-bound state. Apparently, this ensures the correct orientation of the critical carboxy group of Asp-219. The corresponding conserved Asp-205 in PebA is not essential for the conversion of BV to DHBV in PebA (5). Consistently, in PebA no similar coordination network is found, and Asp-205 is pointing away from the substrate (Fig. 4) (5).

Because of the flipped binding mode of DHBV, the distance of Arg-215 to the DHBV A-ring makes a direct involvement in proton transfer impossible. In general, arginine by itself is usually not found to play a role in proton transfer. However, our activity analyses of the variants R215L and R215K point toward a functional role of the arginine. As mentioned above, R215K is able to convert DHBV to PEB with a reduced rate but showing no spectral shift during binding. Because the spectral shifts are proposed to be connected to protonation of the substrate during binding, this suggests that another critical function of the guanidino group of Arg-215 might be the modulation of the p*K_a_* value and therefore the protonation state of the central Asp-99 and/or Asp-219. Indeed, for some members of the lysozyme family, it has been shown that a conserved arginine residue modulates the catalytic capacity of adjacent carboxylates by introducing a strong shift of their p*K_a_* values while also stabilizing the active site structure (24).

### Arg-215 is conserved within A-ring reducing FDBR

It has been postulated that enzymes reducing the A-ring have evolved first and that HY2 is the closest relative to PebB, as based on sequence and function (15, 25). Although the two enzymes bind and process different substrates, they both act on the 2,3,3^1^,3^2^-diene system on the A-ring. Arg-215 (m*Gt*PEBB numbering) indeed is strictly conserved within the complete PebB and HY2 families (Fig. S2 and Refs. 25 and 26). Because there is no structure of HY2 available yet, we generated homology models based on PebA, PebB, or PebS (data not shown). Consistently, these models place the corresponding Arg-252 of HY2 at the identical position in the active site compared with our m*Gt*PEBB structure. Moreover, substitution of Arg-252 in HY2 by glutamine resulted in a complete loss of function for A-ring reduction (26). Therefore, Arg-215 is an essential component of the active site of FDBRs selectively catalyzing A-ring reduction. On a side note, Arg-215 is also conserved in red chlorophyll catabolite reductase from plants, which is distantly related to the FDBR family. There, this residue is pointing out of the active site and not catalytically relevant (27, 28).

### GtPEBB variant R215M can reduce BV

**Figure 6.**
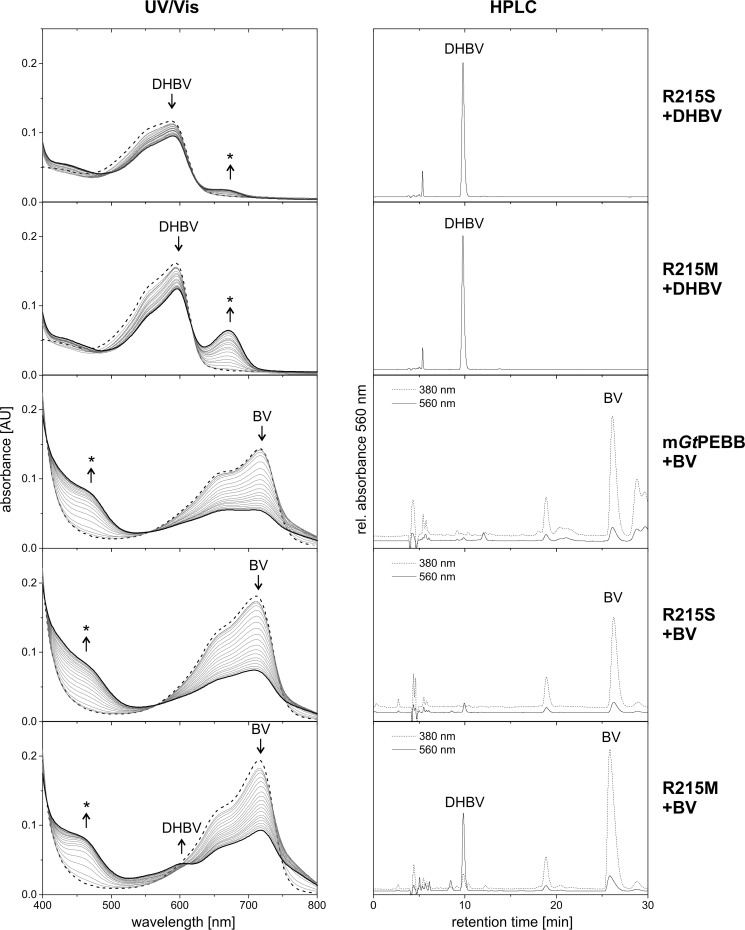
**The reactions of m*Gt*PEBB variants with BV and DHBV were monitored by UV-visible spectroscopy and HPLC analysis of the reaction products.** UV-visible (*UV/Vis*) spectra were taken every 30 s for 10 min. The first spectrum represents the bilin–protein complex and is shown as a *dashed line*. The final spectrum of the reaction is shown in *bold. Arrows* indicate the development of the spectra during the reaction, and possible radical intermediates are marked with *asterisks*. The reaction products after 10 min were analyzed with HPLC monitoring the absorbance at 380 nm (*dotted line*) and at 560 nm (*continuous line*).

Given the critical role of Arg-215 for *Gt*PEBB function, we had a closer look at the corresponding residues in PebA (serine) and PebS (methionine). We therefore analyzed the variants R215S and R215M of m*Gt*PEBB with our standard assay, supplying either DHBV or BV to investigate how these strictly conserved residues are affecting substrate specificity (Fig. 6).

When DHBV was added, both R215S and R215M showed binding spectra similar to WT with a slight shift of the absorbance maximum as already observed for R215L, but no formation of PEB. In the course of the reaction, complex absorption at 594 nm decreased slowly, concomitant with an increase at 670 nm, indicative for radical accumulation. Although this effect was significant in the case of the PebS-like variant R215M, for the PebA-like variant R215S, radical accumulation was considerably reduced. Subsequent HPLC analysis detected only DHBV and no PEB in both cases, indicating a complete loss of DHBV-reductase activity for these two variants.

When we supplied BV to m*Gt*PEBB, the shift of the absorbance maximum to 716 nm (as compared with 681 nm for free BV) and an increase in peak intensity indicated binding of this unnatural substrate. This is in accordance with results reported for the cyanobacterial PebB (11). Similar binding spectra were observed for the variants R215M and R215S. Whereas for WT and R215L only unspecific degradation of BV was detected, R215M was able to convert BV to DHBV. This was indicated by a slight increase of the absorbance at 605 nm and was verified by HPLC analysis of the reaction products. Therefore, the PebS-like variant R215M acquired the ability to reduce the C15–C16 double bond of BV with low efficiency, yet lost the ability to catalyze the A-ring reduction, which is done in the second step by PebS.

Because we do not yet have information on the binding mode of BV in PebB or PebB variants or on the binding mode of DHBV in PebS, further experimental data are needed. At least this suggests that PebS utilizes a different molecular mechanism for the A-ring reduction during the conversion of DHBV to PEB than PebB.

### A primary site for two-electron reduction

Taken together, the available data indicate that in FDBRs catalyzing a two-electron reduction, this reaction will always take place in the same region of the binding pocket (on the left of the central aspartates in Fig. 4, termed primary site). Accordingly, this primary site is occupied either by the D-ring in the case of PebA or by the A-ring in the case of PebB. For PebA, a reaction mechanism consistent with this geometry was put forward (5).

For PebB, the following reaction mechanism would be consistent with our structural and biochemical data of variants (Table 2). After binding of DHBV to *Gt*PEBB, Asp-99 delivers a proton to the A-ring oxygen, forming a positively charged DHBVH+. After acceptance of an electron from ferredoxin, the A-ring pyrrole proton tautomerizes to the C2 position and is stabilized there. This is facilitated by the catalytic action of the axial water, which is activated/positioned by Asp-219 and (indirectly) by Arg-215. For the second protonation step, it remains unclear which molecular determinants govern the protonation of the vinyl group at the C1 position. One possible scenario is the reprotonation of the A-ring nitrogen, uptake of another electron, and a final tautomerization to yield the product PEB. Again, the position of the axial water makes it a likely determinant for the stereospecificity of this step.

**Table 2:** **Activities of mGtPEBB wildtype and variants** NA, not available; +, wildtype reference; ∼, moderate; –, only trace amounts; —, not detectable.

m*Gt*PEBB variant	DHBV		BV		
	Spectral shift/binding	Conversion to PEB	Spectral shift/binding	Conversion	
WT	+	+	+	–	
D99N	∼	—	NA	NA	Critical residue
D219N	∼	—	NA	NA	Critical residue
R215A	∼	–	NA	NA	Critical residue
R215L	+	—	NA	NA	
R215K	∼	∼	NA	NA	
R215S	∼	—	+	–	Conserved in PebA
R215M	+	—	+	∼ (DHBV)	Conserved in PebS

Interestingly, this proposed mechanism would be in agreement with the biochemical data available for HY2, which catalyzes the identical reduction on the A-ring (but on BV instead of DHBV) (26). In addition, this is fully consistent with the close placement of both proteins in evolutionary trees (25). Although we still do not have structural data for binding of BV in HY2, the conservation of critical active site residues discussed above would suggest the placement of the A-ring in the primary site. It is interesting to note, that HY2 from vascular plants is the only FDBR in which the central aspartate Asp-99 is not conserved but replaced by an asparagine. Although this residue has been shown to be involved in substrate binding, its critical role in FDBR activity has been replaced by Asp-116 in HY2 (26). In all homology models, this residue would be in a similar distance to the pyrrole ring oxygen in the primary site, functionally replacing Asp-99 in our *Gt*PEBB mechanistic model outlined above. This again supports a binding mode of BV with the A-ring in the primary site in the case of HY2. Even more interesting, HY2 from ferns or mosses have retained the canonical aspartate pair, supporting their placement close to the base of the evolutionary tree of FDBR

### Consequences of flipped binding mode for PEB biosynthesis

The flipped binding mode of DHBV in *Gt*PEBB raises the question of whether BV conversion to PEB functions in the same mechanistic manner in the PebA/PebB system and in PebS. Available crystal structures show BV bound in the same orientation in both PebA and PebS, leading to the conclusion that they share the reaction mechanism for the first two-electron reduction on the D-ring. This is supported by the biochemical data.

If A-ring reduction in PebS works in the same mechanistic manner as in PebB, DHBV would have to be released after the first reduction and bound again in the flipped orientation. One can speculate whether this would be realized by a metabolic channeling between two PebS molecules, as described for PebA and PebB (11, 12).

Until now, there is no evidence that the intermediate DHBV is released from PebS after the first reduction. Because the binding niche of PebS provides somewhat more space than in PebA, this would allow a rearrangement of the intermediate DHBV but would still retain the placement of the A-ring in the second site (on the right of the central aspartate pair) (5). Clearly, this would require a reaction mechanism distinct from the one outlined above. At the same time, PcyX catalyzes the same reaction as PebS but is more closely related to PcyA and lacks essential residues of PebS. This example shows that the same reaction can be facilitated by different molecular mechanisms within the FDBR family. Because substrate orientation is fundamental for these mechanistic questions, a crystal structure of PebS with bound intermediate DHBV would be highly desirable.

## Experimental procedures

### Chemicals

All chemicals were ACS grade or better unless specified otherwise. The Assay components including BV were purchased from Sigma–Aldrich.

### Construction of expression plasmids and site-directed mutagenesis

The genome-derived sequence of *Gt*PEBB contains a predicted signal peptide of 18 residues and a predicted targeting sequence including residues 19–83. The protein without the signal peptide is referred to as *Gt*PEBB (for consistency with the published construct) (19). The predicted mature protein m*Gt*PEBB starts at residue 84 and does not contain any targeting signal. Plasmids for heterologous protein expression of *Gt*PEBB and m*Gt*PEBB in *Escherichia coli* were constructed with Gibson Assembly® (New England Biolabs) according to the manufacturer's instructions. The vector pGEX-4T-1 (GE Healthcare) with an additional tobacco etch virus (TEV) protease recognition site was linearized by PCR amplification. The plasmid p*GtPEBB* (codon optimized gene, without signal peptide) (19) was used as a template to amplify the inserts with overlapping ends by PCR applying the primers listed in Table S2. The resulting fragments were separated by agarose gel electrophoresis, extracted from appropriate gel bands with the NucleoSpin® gel and PCR clean up kit (Macherey–Nagel) before ligation by the Gibson Assembly Master Mix and subsequent transformation into NEB® 5-alpha competent *E. coli* cells. The resulting constructs contain *Gt*PEBB/m*Gt*PEBB as translational fusion to GST with a TEV site following the thrombin site.

All variants of m*Gt*PEBB were constructed by PCR site-directed mutagenesis using the primers listed in Table S3. Because the reverse primer is complement, only the forward primer is shown. Introduced bp changes are underlined. Variants R215A, R215L, and R215K were constructed using the QuikChange® Lightning kit (Agilent) according to the manufacturer's protocol. PCRs for R215M, R215S, D99N, and D219N were set up using the *PfuUltra* II Fusion HS polymerase (Agilent). After DpnI digestion, aliquots of the reaction were transformed into NEB® 5-alpha competent *E. coli* cells. All resulting plasmids were checked by sequencing.

### Production and purification of recombinant proteins

For recombinant production of *Gt*PEBB and m*Gt*PEBB and variants, 2× YT medium containing 100 μg/ml ampicillin was inoculated 1:100 with an overnight preculture carrying the respective expression plasmid. 1-liter cultures were grown in a 5-liter flask at 37 °C and 100 rpm to an *A*_600_ of 0.7 followed by a temperature shift to 30 °C before induction with 300 μm isopropyl-β-d-thiogalactopyranosid. After 4 h expression, the cells were harvested by centrifugation (6000 × *g*, 12 min) and stored at −20 °C for further use. Thawed pellets were homogenized in 10 ml of lysis buffer (50 mm Tris, pH 7.5, 300 mm NaCl) per gram of cells, and a spatula tip of DNase I (Sigma–Aldrich) was added prior to cell disruption with a microfluidizer M-100L (Microfluidics) at 13,000 p.s.i. After ultracentrifugation (220,000 × *g*, 1 h, 4 °C), the supernatant was filtered through a 0.2-μm filter and loaded onto a PureCube GSH-agarose (Cube Biotech) column. After washing, bound protein was eluted with elution buffer (50 mm Tris, pH 7.5, 300 mm NaCl, 10 mm l-GSH reduced). Cleavage of the fusion protein was done with a GST-tagged TEV protease (1 mg of GST-TEV per 50 mg of fusion protein) overnight during dialysis against a 200-fold volume of lysis buffer in a 6,000–8,000-Da molecular mass cutoff tubing (Spectrum Labs). The protease digest produced the protein of interest with three additional amino acids at the N terminus (Gly, Gly, Ser) or two additional N-terminal amino acids (Gly, Gly) for m*Gt*PEBB. To remove residual GST and GST-TEV, the dialysate was loaded again on the GST affinity column. The flow through was collected and run on a HiLoad^TM^ 16/600 Superdex^TM^ 75-pg (GE Healthcare) size exclusion column equilibrated with low-salt buffer (20 mm Tris, pH 7.5, 50 mm NaCl) for crystallization experiments or 25 mm TES/KOH, pH 7.5, 100 mm KCl for activity assays. All chromatography steps were carried out on an ÄKTA purifier 100 system (GE Healthcare). The protein was concentrated using Vivaspin® centrifugal concentrators with a 10,000-Da molecular mass cutoff (Sartorius AG).

PebA from *Synechococcus* sp. WH8020 was expressed and purified as described before with slight modifications (11). The cells were resuspended in 50 mm Tris, pH 8.0, 150 mm NaCl, 5 mm MgCl_2_. Cell disruption with a microfluidizer M-100L (Microfluidics) instead of a French press and ultracentrifugation were done as described above for PebB. For GST affinity chromatography PureCube GSH-agarose resin (Cube Biotech) was used. Final buffer exchange was done by gel filtration on a HiLoad^TM^ 16/600 Superdex^TM^ 75-pg column (GE Healthcare) instead of dialysis. Prior to snap freezing PebA in liquid N_2_ for storage at −80 °C, the solution was supplemented with 10% glycerol. Ferredoxin from *Synechococcus* sp. PCC7002 was expressed and purified as described in Ref. 11.

### Selenomethionine–GtPEBB for experimental phasing

For expression of selenomethionine containing *Gt*PEBB the standard protocol described for *Gt*PEBB was modified according to Refs. 29 and 30. The cells were grown in M9 minimal medium supplemented with vitamins and trace elements. 15 min prior to induction, a feedback inhibition amino acid mix (lysine, phenylalanine, threonine: 100 mg/liter; valine, leucine, isoleucine, l(+)-selenomethionine: 50 mg/liter) was added to the culture. All buffers used during purification contained 10 mm DTT.

### Crystallization of GtPEBB

Crystallization conditions for *Gt*PEBB were screened by the sitting-drop vapor-diffusion method applying 100/100- and 200/100-nl mixtures of protein solution (7–10 mg/ml)/reservoir solution incubated at 18 and 4 °C in the dark. Initial crystals grew in 0.1 m Tris pH 8.5, 35% PEG 3350, and 0.2 m ammonium sulfate (MemGold2–E5; Molecular Dimensions) at 4 °C. Final crystals of selenomethionine substituted *Gt*PEBB were obtained by optimization of the initial condition of the native protein by the hanging-drop vapor-diffusion method in a 2/1-μl drop of 7 mg/ml protein and 0.1 m Tris, pH 8.5, 34.5% PEG 3350, 0.21 m ammonium sulfate reservoir solution after 4 days at 4 °C.

Crystallization of m*Gt*PEBB was optimized, and final crystals grew in a 1/1-μl hanging drop over a reservoir solution containing 0.1 m Tris, pH 8.5, 34.5% PEG 3350, 0.15 m ammonium sulfate. For crystallization of the m*Gt*PEBB–DHBV complex, protein solution was incubated with a 2-fold molar excess of DHBV dissolved in DMSO for 1 h on ice in the dark. The complex and free DHBV were separated by applying to a NAP^TM^-5 column (GE Healthcare). To achieve stabilization of DHBV during crystal growth, sitting-drop grid screen refinement plates were resealed and stored in an anaerobic chamber CLc (Coy Laboratory Products) after setting them up under standard conditions. Final purple colored crystals grew in a 200/100-nl mixture of protein (8 mg/ml)/reservoir (0.1 m Tris, pH 8.5, 32.5% PEG 3350, 0.23 m ammonium sulfate) at 6 °C in the dark. Prior to freezing in liquid N_2_, all crystals were briefly soaked in mother liquor supplemented with 16% PEG 400 for cryoprotection.

### Data collection and structure determination

Oscillation data were collected at 100 K at the Swiss Light Source (Villigen, Switzerland) at Beamline PXII–X10SA and at the European Synchrotron Radiation Facility (Grenoble, France) at Beamline ID30B using PILATUS 6M detectors (DECTRIS). All data were processed and scaled using XDS and XSCALE (31). The data set of substrate-bound m*Gt*PEBB was radiation damage corrected by zero-dose extrapolation in XSCALE. Statistics of all data sets are listed in Table 1.

Because molecular replacement using several FDBRs as a search model failed, phases were determined from a 2.5 Å data set of selenomethionine-labeled protein, collected at 0.97625 Å (Se-K edge). For high redundancy and low dose, the data were collected covering 720° rotation range. Ten selenium sites were refined with Phenix.autosol (32) of which nine were present in the final model. We could autotrace 244 residues of *Gt*PEBB in 7 fragments with Phenix.autobuild (32). This initial model was subsequently improved using Coot (33) and Phenix.refine (32), but clear density for ∼70 N-terminal amino acids was missing.

The initial model of selenomethionine labeled *Gt*PEBB was then used for refinement against the native 1.9 Å data set of m*Gt*PEBB, which was obtained by scaling two data sets from individual crystals. The final model of the substrate-free protein was deposited in the PDB with the accession code 6QWQ. Because of weak or missing density, the first three residues and residues 45–48 and 147–151 have been omitted.

Because the m*Gt*PEBB–DHBV complex crystallized in a different space group, we used the substrate-free structure as a search model for molecular replacement with Phenix.phaser (32). Geometry restraints for DHBV were calculated using Phenix.elbow (32). The model of the substrate loaded protein was again improved using Coot (33) and Phenix.refine (32) and was deposited in the PDB with accession code 6QX6. In the final model, the first six residues (first four in case of chain A), residues 44–49 and the last residue were not modeled because of missing density. Because the overall quality of chain B (including a fully occupied DHBV) was better than that of chain A (with a partially occupied binding pocket, DHBV occupancy: 0.75), we used chain B for all figures.

### Bilin reductase activity test

Anerobic bilin reductase assays were performed as described before with slight modifications (21). Assay conditions consisted of 25 mm TES/KOH, pH 7.5, 100 mm KCl, 10 μm FDBR, 10 μm substrate (BV/DHBV), 10 μm BSA, 100 mm glucose, 50 units/ml glucose oxidase, 50 units/ml catalase, 1 μm ferredoxin from *Synechococcus* sp. PCC7002, and 0.01 μm ferredoxin NADP^+^ reductase from *Synechococcus* sp. PCC7002. Catalysis was started by the addition of 100 μl of a NADPH regenerating system (65 mm glucose-6-phosphate, 8.2 mm NADP^+^, 11 units/ml glucose-6-phosphate dehydrogenase), ending up with a final assay volume of 2 ml (14).

Utilizing a 8453 series diode array spectrophotometer (Agilent), spectra were taken every 30 s for 10 min. The reaction was stopped by the addition of a 10-fold volume of 0.1% TFA. Bilins were extracted from the reaction mixture using C18 Sep-Pak light cartridges (Waters), which were preconditioned as described before (11). After elution with acetonitrile, bilins were freeze-dried using an Alpha 2-4 LSC plus lyophilizer (Martin Christ GmbH) for subsequent HPLC analyses.

### HPLC analyses

HPLC analyses were performed as described before, using an 1100 series HPLC system (Agilent) with a Luna 5μ C18 reversed-phase column (Phenomenex) and 50% acetone and 50% 20 mm formic acid as the mobile phase (6).

### Enzymatic production of DHBV

PebA from *Synechococcus* sp. WH8020 was used for enzymatic production of DHBV for *Gt*PEBB activity tests and co-crystallization experiments. To obtain higher yields, the activity assay was modified by feeding 2–3× new BV after no residual BV was detectable by UV-visible spectroscopy (17). The eluate of C18 Sep-Pak light cartridges (Waters) was further purified by HPLC using a Smartline 1800 preparative HPLC system (Knauer) with a Eurospher II 100-5 200 × 20-mm C18 reversed-phase column (Knauer) and 30% acetonitrile in 50 mm P_i_, pH 6.5, as the mobile phase. Fractions with pure DHBV were again loaded on a Sep-Pak light cartridge before lyophilization. The lyophilizate was dissolved in DMSO for further experiments.

### pK_a_ calculations

Protonation states were assigned by predicting the p*K_a_* values of titratable groups with the residue p*K_a_* application included in the Molecular Operating Environment platform with default settings (34). Residue p*K_a_* is based on PROPKA, but modifications allow that the calculation is not restricted to titratable groups of standard amino acid side chains (34, 35).

## Author contributions

J. A. S., N. F.-D. and E. H. conceived and designed the study. J. A. S. performed biochemical experiments, crystallized the proteins, and solved the structures. J. A. S. and E. H. analyzed the structural data. J. A. S., N. F.-D., and E. H. wrote the manuscript and agreed on the final version of the manuscript.

## Supplementary Material

Supporting Information

## Footnotes

2 The abbreviations used are:

BV: biliverdin
DHBV: dihydrobiliverdin
FDBR: ferredoxin-dependent bilin reductase
HY2: phytochromobilin synthase
PcyA: phycocyanobilin:ferredoxin oxidoreductase
PEB: phycoerythrobilin
PebA: 15,16-dihydrobiliverdin:ferredoxin oxidoreductase
PebB: PEB:ferredoxin oxidoreductase
PebS: phycoerythrobilin synthase
PDB: Protein Data Bank
TEV: tobacco etch virus
GST: glutathione *S*-transferase.
