# Supplementary material for: Crystal structure of the first eukaryotic bilin reductase GtPEBB reveals a flipped binding mode of dihydrobiliverdin
Source: J Biol Chem. 2019 Jul 31;294(38):13889–901. doi: 10.1074/jbc.RA119.009306 (PMC6755814; doi:10.1074/jbc.RA119.009306)
Supplement: Supporting Information [file supp_294_38_13889__index.html]

Crystal structure of the first eukaryotic bilin reductase GtPEBB reveals a flipped binding mode of dihydrobiliverdin — Structure of GtPEBB — Crystal structure of the first eukaryotic bilin reductase GtPEBB reveals a flipped binding mode of dihydrobiliverdin — Structure of GtPEBB — Supporting Information 

# Crystal structure of the first eukaryotic bilin reductase *Gt*PEBB reveals a flipped binding mode of dihydrobiliverdin

## Supporting Information

- Supporting Information (to be published online) - Supporting figures and tables
