# Supplementary material for: Crystal structure of the first eukaryotic bilin reductase GtPEBB reveals a flipped binding mode of dihydrobiliverdin
Source: J Biol Chem. 2019 Jul 31;294(38):13889–901. doi: 10.1074/jbc.RA119.009306 (PMC6755814; doi:10.1074/jbc.RA119.009306)
Supplement: Supporting Information [file supp_RA119.009306_152758_1_supp_369421_pv974y.pdf]

Crystal structure of the first eukaryotic bilin reductase *Gt*PEBB reveals a flipped binding mode of dihydrobiliverdin

Johannes A. Sommerkamp[1], Nicole Frankenberg-Dinkel[2] and Eckhard Hofmann[1,*]

[1] Protein Crystallography, Faculty of Biology and Biotechnology, Ruhr University Bochum, Germany
[2] Department of Biology, Microbiology, Technical University Kaiserslautern, Germany
*Corresponding author: email: eckhard.hofmann@rub.de; phone: +49 234 3224463

Supporting Information

Table S1: RMSDs between different FDBRs

Table S2: Primers used for the construcion of the expression plasmids

Table S3: Primers used for site-directed mutagenesis

Figure S1: Slight conformational changes upon substrate binding

Figure S2: Sequence-alignment of various members of the FDBR family

Figure S3: Stereo views of the active sites of PebA, PebB and PebS

Figure S4: Active site of m*Gt*PEBB-DHBV (6QX6) chain A

Table S1. RMSDs between different FDBRs.

**m*Gt*PEBB-DHBV (chainB) vs.**	**RMSD [Å] PyMOL super (Cα only)**
PebA-BV (2X9O)	1.535 (186 atoms)
PebS-BV (2VCK chain C)	1.796 (137 atoms)
PcyA-BV (2D1E)	2.273 (195 atoms)
PcyX (5OWG) without substrate	2.243 (139 atoms)

Table S2. Primers used for the construcion of the expression plasmids. Non-matching overlaps in lower case.

Overlaps	Sequence 5' to 3'	Fwd/Rev	Anneals	Resulting Plasmid
-	GAATTCCCGGGTCGACTC	Fwd	pGEX-4T-1-TEV	Linearization
-	ggatcCGCCCTGAAAATAAAGGTTTTCAGATCCAC	Rev	pGEX-4T-1-TEV	Linearization
pGEX-4T-1-TEV	tttcagggcggatccTTTCAGTTAGGTACTCCGG	Fwd	*GtPEBB*	pGEX-4T-1-TEV_*GtPEBB*
pGEX-4T-1-TEV	tcgacccgggaattcTTATAAGGGCGTTGGTTG	Rev	*GtPEBB*	pGEX-4T-1-TEV_*GtPEBB*
pGEX-4T-1-TEV	tttcagggcggatccGAAGCGGTATCTGACATC	Fwd	*GtPEBB*	pGEX-4T-1-TEV_*mGtPEBB*
pGEX-4T-1-TEV	tcgacccgggaattcTTATAAGGGCGTTGGTTG	Rev	*GtPEBB*	pGEX-4T-1-TEV_*mGtPEBB*

Table S3. Primers used for site-directed mutagenesis.

Variant	Sequence 5' to 3'
R215A	GAGCTATCTGCAGTATGCGGCCGAGAAAGATCCTG
R215L	GCTATCTGCAGTATCTGGCCGAGAAAGATCC
R215K	CTGAGCTATCTGCAGTATAAAGCCGAGAAAGATCCTGC
R215M	GAGCTATCTGCAGTATATGGCCGAGAAAGATCCTG
R215S	GCTATCTGCAGTATAGCGCCGAGAAAGATCC
D99N	CTTTCCTGGGCCTGAACCTGGTCACCCTG
D219N	GTATCGCGCCGAGAAAAACCCTGCTCGTGGAATG

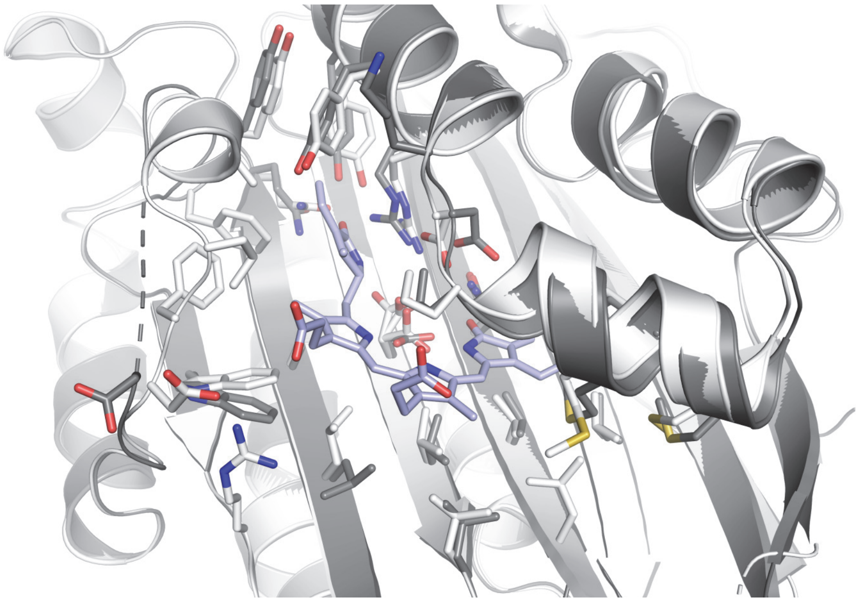

Figure S1. Slight conformational changes upon substrate binding. Superposition of substrate free m*Gt*PEBB (dark grey, PDB-code: 6QWQ) and m*Gt*PEBB with bound DHBV (light grey, PDB-code: 6QX6. DHBV is shown in stick representation (purple). The loop region forming the lid of the active site could not be resolved in the substrate free structure (indicated by a dotted line).

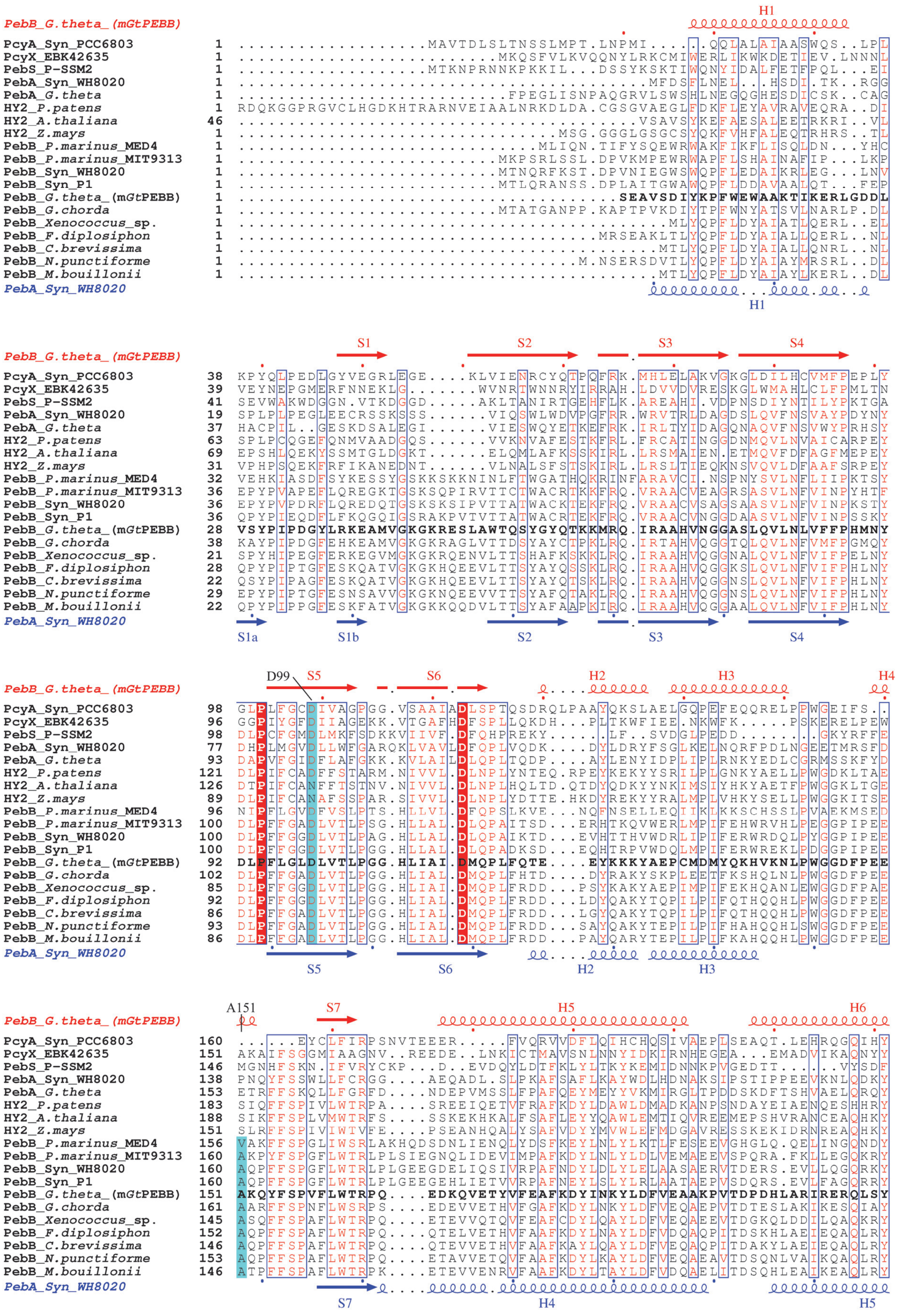
H1
S1
S2
S3
S4
S1a
S1b
D99
S5
S6
H2
H3
H4
A151
S7
H5
H6

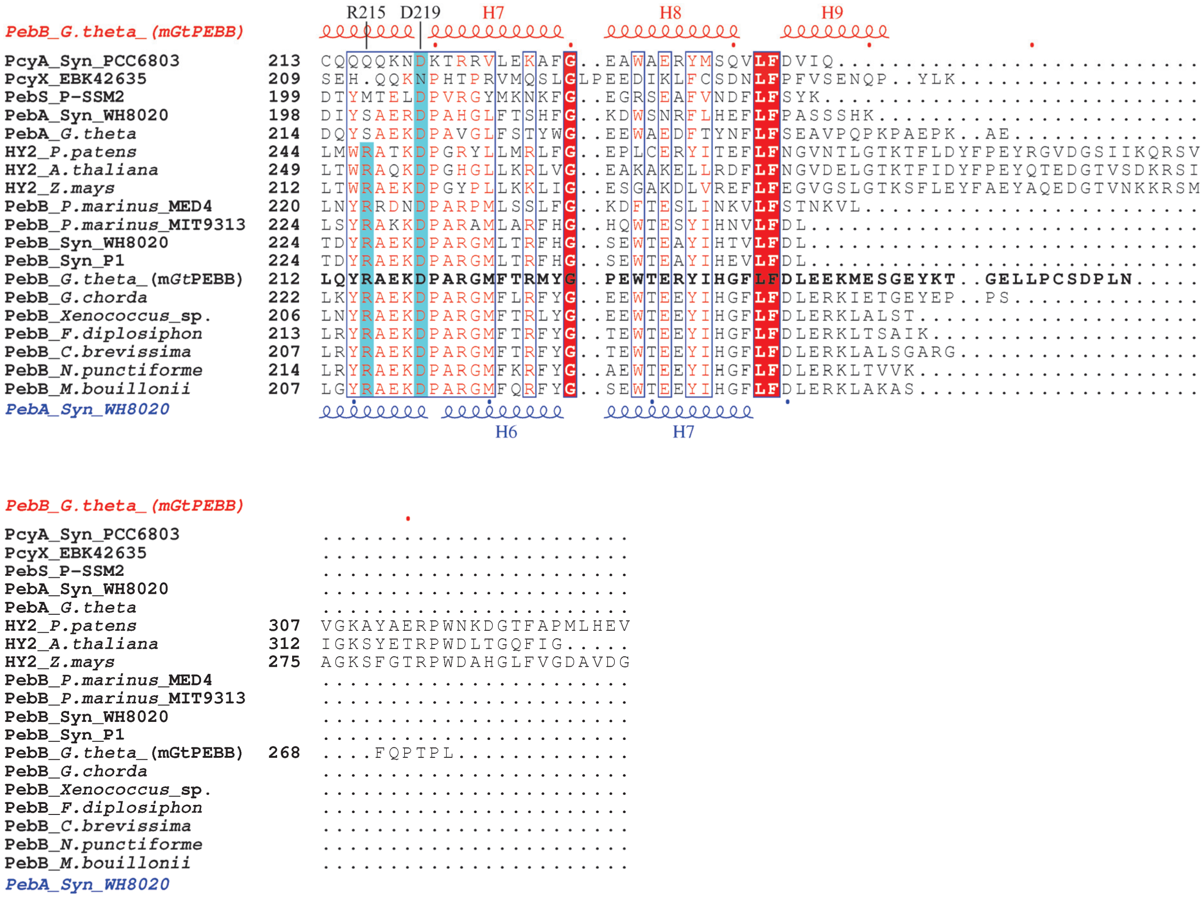

Figure S2. Sequence-alignment of various members of the FDBR family. Sequences were selected for which biochemical or structural data are available. Additionally, several PebB sequences were selected to cover different domains of a phylogenetic tree. Sequences were aligned using Clustal-Omega (1) and ESPript (2) was used for graphical representation. Residues discussed in the main text are highlighted in cyan and labeled according m*Gt*PEBB numbering. Sequences of eukaryotic organisms do not contain predicted signal peptides / transit sequences. Numbering starts with the first amino acid of the mature protein, except for HY2 from *A. thaliana* we adopted the numbering from reference (3). Secondary structure assignment is based on m*Gt*PEBB (6QX6) and PebA (2X9O) and matches to Fig. 2A.

PcyA_Syn_PCC6803: PcyA from *Synechocystis* sp. PCC6803, Q55891
PcyX_EBK42635: PcyX, hypothetical protein GOS_8734801 [marine metagenome], EBK42635
PebS_P-SSM2: PebS from *Prochlorococcus* phage P-SSM2, Q58MU6
PebA_Syn_WH8020: PebA from *Synechococcus* sp. WH8020, Q02189
PebA_*G.theta*: PebA from *Guillardia theta*, AIA66935.1
HY2_*P.patens*: HY2 from *Physcomitrella patens,* BAF02520.1
HY2_*A.thaliana*: HY2 from *Arabidopsis thaliana*, Q9SR43
HY2_*Z.mays*: HY2 from *Zea mays,* AAT66259.1
PebB_*P.marinus*_MED4: PebB from *Prochlorococcus marinus* MED4, AAK38142.1
PebB_*P.marinus*_MIT9313: PebB from *Prochlorococcus marinus* MIT9313, CAE21862.1
PebB_Syn_WH8020: PebB from *Synechococcus* sp. WH8020, AAK77916.1
PebB_Syn_P1: PebB from *Synechococcus* sp. P1, WP_115022899.1
PebB_*G.theta*_(m*Gt*PEBB): PebB from *Guillardia theta*, AIA66937.1
PebB_*G.chorda*: PebB from *Gracilariopsis chorda*, PXF47564.1
PebB_*Xenococcus*_sp.: PebB from *Xenococcus* sp. PCC7305, WP_006511008.1
PebB_*F.diplosiphon*: PebB from *Fremyella diplosiphon*, AAQ63054.1
PebB_*C.brevissima*: PebB from *Calothrix brevissima*, WP_096646461.1
PebB_*N.punctiforme*: PebB from *Nostoc punctiforme*, AAK38589.1
PebB_*M.bouillonii*: PebB from *Moorea bouillonii*, WP_075904181.1

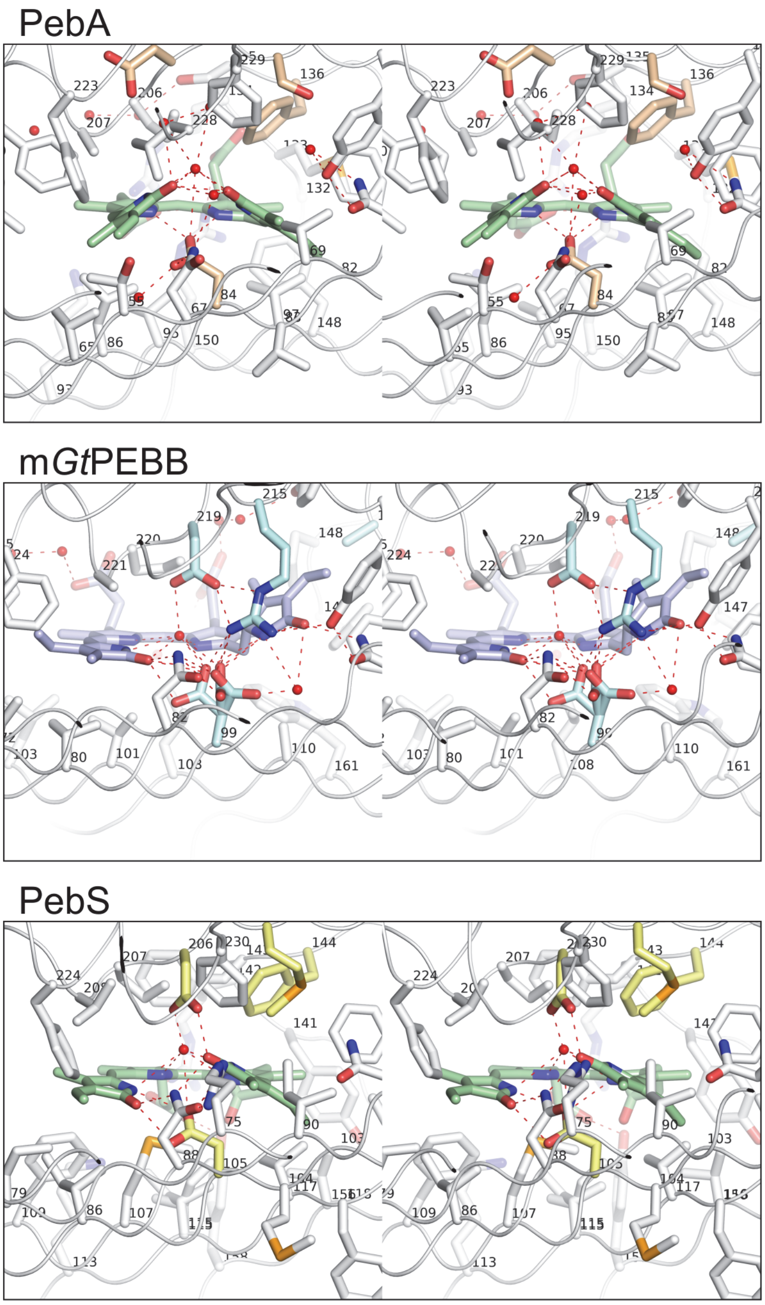

Figure S3. Stereo views of the active sites of PebA, PebB and PebS. Active sites of PebA (2X9O), m*Gt*PEBB (6QX6, chain B) and PebS (2VCK, chain C) with their specific substrate BV (green) and DHBV (purple) in stick representation. BV of PebA and PebS is oriented with the D-ring left and the A-ring right whereas DHBV bound to PebB in a flipped mode is oriented with the D-ring right and A-ring left. Residues in 4.5 Å distance around the substrate are represented as sticks, additionally residues discussed in the text are highlighted in color. Water molecules are shown as red spheres and red dotted lines represent polar contacts.

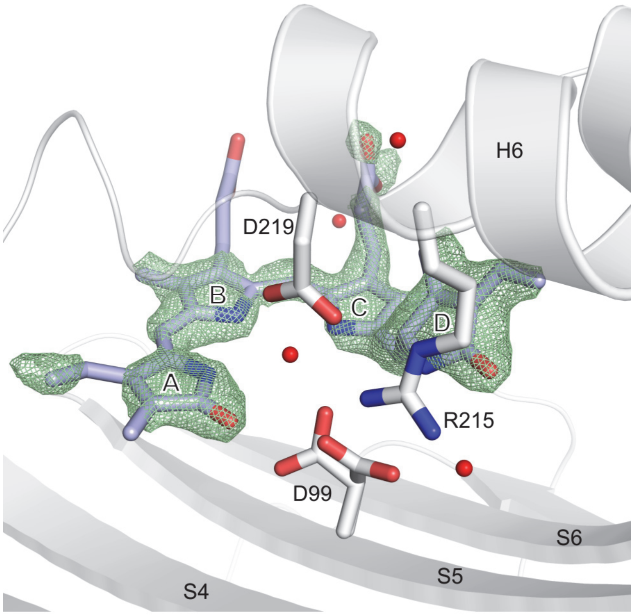

Figure S4. Active site of m*Gt*PEBB-DHBV (6QX6, chain A). DHBV (purple) and residues critical for the PebB reaction are represented as sticks. Water molecules in 4 Å distance to DHBV are shown as red spheres. A simulated annealing OMIT-map for DHBV is shown as a green mesh, contoured at 0.9 σ.

Supporting References

1. Sievers, F., Wilm, A., Dineen, D., Gibson, T. J., Karplus, K., Li, W., Lopez, R., McWilliam, H., Remmert, M., Söding, J., Thompson, J. D., and Higgins, D. G. (2011) Fast, scalable generation of high-quality protein multiple sequence alignments using Clustal Omega. *Mol. Syst. Biol.* **7**, 539
2. Robert, X., and Gouet, P. (2014) Deciphering key features in protein structures with the new ENDscript server. *Nucleic Acids Res.* **42**, W320–W324
3. Tu, S.-L., Chen, H.-C., and Ku, L.-W. (2008) Mechanistic studies of the phytochromobilin synthase HY2 from Arabidopsis. *J. Biol. Chem.* **283**, 27555–27564
